# Kaempferol Promotes Apoptosis in Human Bladder Cancer Cells by Inducing the Tumor Suppressor, PTEN

**DOI:** 10.3390/ijms141121215

**Published:** 2013-10-24

**Authors:** Feng Xie, Ming Su, Wei Qiu, Min Zhang, Zhongqiang Guo, Boxing Su, Jie Liu, Xuesong Li, Liqun Zhou

**Affiliations:** 1 Department of Urology, Peking University First Hospital & the Institute of Urology, Peking University, Beijing 100034, China; 2 National Urological Cancer Center, Beijing 100034, China; 3 Sino-German Laboratory for Molecular Medicine, State Key Laboratory of Cardiovascular Disease, FuWai Hospital & Cardiovascular Institute, Chinese Academy of Medical Sciences, Peking Union Medical College, Beijing 100037, China; 4 Department of Urology, Shanxi Medical University First Hospital, Taiyuan 030001, China

**Keywords:** kaempferol, bladder cancer, apoptosis, PTEN, Akt

## Abstract

Kaempferol (Kae), a natural flavonoid, is widely distributed in fruits and vegetables. Previous studies have identified Kae as a possible cancer preventive and therapeutic agent. We found Kae to exhibit potent antiproliferation and anti-migration effects in human bladder cancer EJ cells. Kaempferol robustly induced apoptosis in EJ cells in a dose-dependent manner, as evidenced by increased cleavage of caspase-3. Furthermore, we found Kae-induced apoptosis in EJ cells to be associated with phosphatase and the tensin homolog deleted on the chromosome 10 (PTEN)/PI3K/Akt pathway. Kae significantly increased PTEN and decreased Akt phosphorylation. Kae-induced apoptosis was partially attenuated in PTEN-knockdown cells. Our findings indicate that Kae could be an alternative medicine for bladder cancer, based on a PTEN activation mechanism.

## 1. Introduction

Bladder cancer is one of the most common cancers in the world [1]. Although current treatments for bladder cancer—surgery, radiation therapy, chemotherapy or their combination—prolong survival time, bladder cancer tends to recur and progress. Thus, there is an urgent need for developing a novel treatment for bladder cancer.

Abundant intake of fruits and non-starchy vegetables has been proposed as a beneficial lifestyle factor for the prevention of a wide variety of cancer types [2]. Kaempferol (Kae) is a natural flavonoid, widely found in fruits and vegetables, including strawberries, broccoli, tea and red grapes [3,4], and accounts for 25%–33% of the mean dietary flavonol intake in the USA [5]. The anticarcinogenic, cardioprotective and anti-aging properties of Kae have received much attention [6–8], as has the evidence that it inhibits various human cancer cell lines, such as non-small-cell lung cancer, ovarian cancer, leukemia, prostate cancer, breast cancer and colon cancer [9–14]. However, the effects of Kae on bladder cancer remain unclear. In this investigation of the antitumor effects of Kae on human bladder cancer cells, we found that Kae increases apoptosis and decreases invasion in human bladder cancer EJ cells. Phosphatase and tensin homolog deleted on chromosome 10 (PTEN) is a potent tumor-suppressor gene. Loss of functional PTEN is frequently observed in bladder cancers [15] and is associated with chemotherapy resistance [16,17]. PTEN is critical in cell proliferation and apoptosis, predominantly through dephosphorylation of phosphatidylinositol (3,4,5)-trisphosphate (PIP3) and inhibition of the protein kinase B (Akt) signaling pathway [18]. Our results indicate that Kae induces apoptosis in bladder cancer EJ cells by affecting the PTEN/phosphatidyl inositol-3-kinase (PI3K)/Akt pathway. Kaempferol significantly increases PTEN expression and decreases Akt phosphorylation, which potentiates its antitumor effects in EJ cells.

## 2. Results and Discussion

### 2.1. Results

#### 2.1.1. Kae Inhibits the Viability of Bladder Cancer Cells

**Figure 1:**
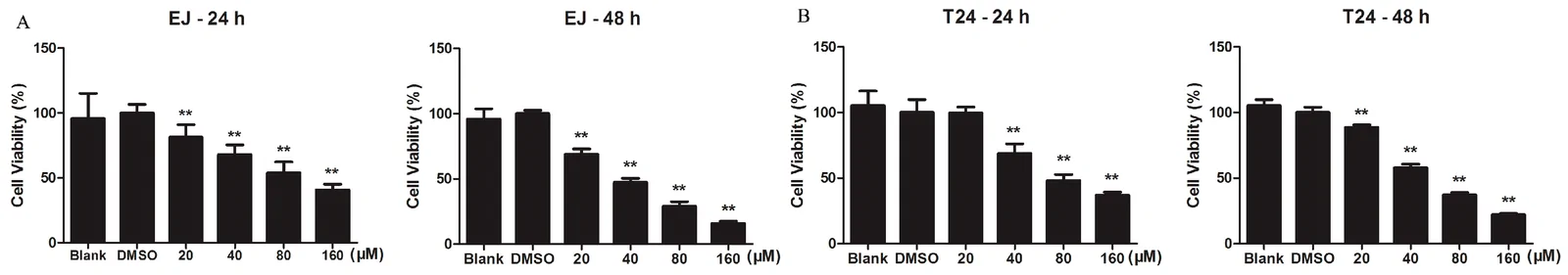
Kaempferol (Kae) inhibits viability of EJ (**A**) and T24 cells (**B**). Cells were treated with DMSO or indicated concentrations of Kae for 24 h and 48 h. Cells without any treatment were indicated as blank. Cell viability was determined by Cell Counting Kit-8 (CCK-8) assay. Data shown are the mean ± SD of three independent experiments. **p < 0.01 *vs*. DMSO-only group.

To find Kae’s effects on cell viability, we performed a Cell Counting Kit-8 (CCK-8) assay on bladder cancer EJ and T24 cells treated with Kae (20, 40, 80 or 160 μM) or dimethyl sulfoxide (DMSO, vehicle) for 24 h and 48 h. Kae was found to significantly inhibit the growth of EJ and T24 cells in a dose-dependent manner (Figure 1). Calculated half maximal inhibitory concentration (IC_50_) values were 78.4 and 38.1 μM in EJ cells and 85.3 and 54.2 μM in T24 cells at 24 h and 48 h of treatment, respectively. EJ cells were significantly inhibited by Kae at 20 μM after 24 h treatment (*p* < 0.01) and were more sensitive to Kae compared to T24 cells.

#### 2.1.2. Kae Induces Apoptosis by Activating the Caspase-3 Pathway in EJ Cells

**Figure 2:**
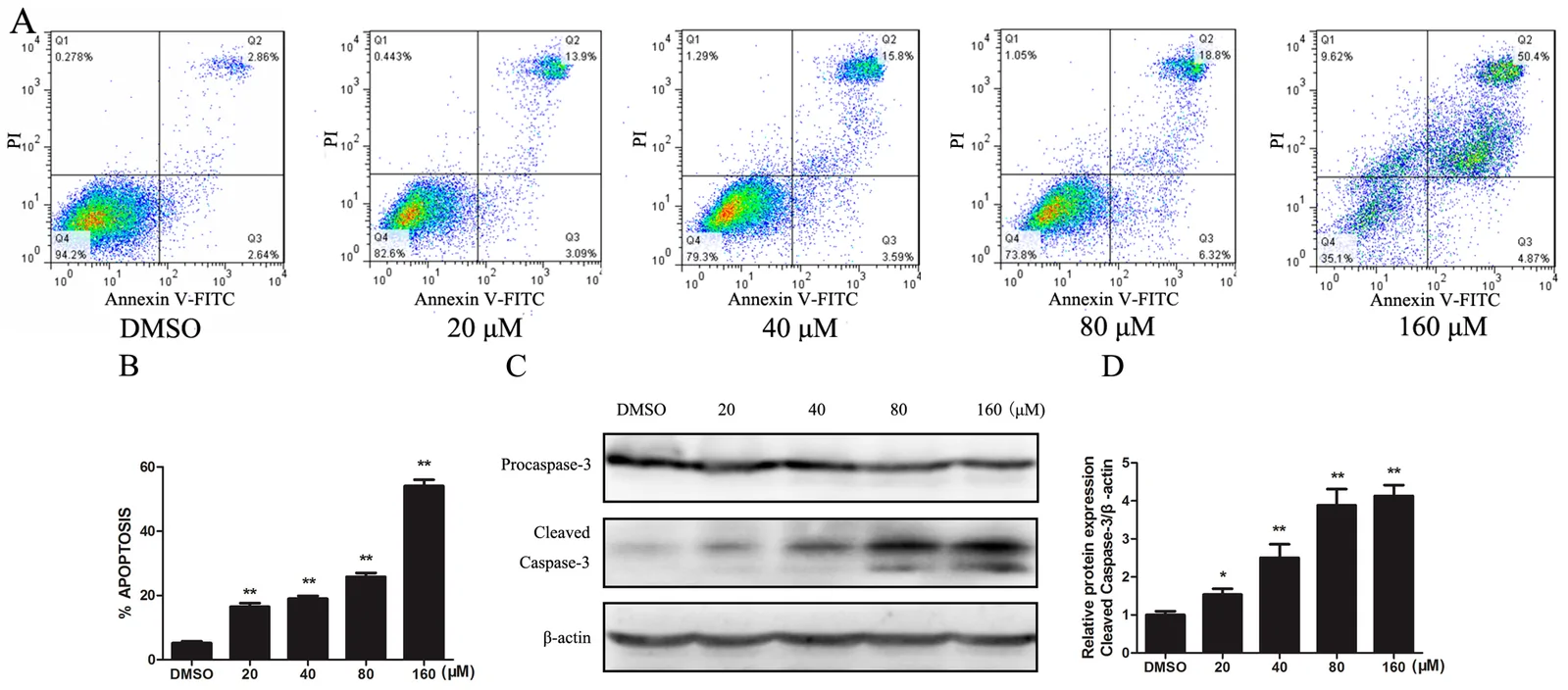
Kae induces apoptosis by activating the caspase-3 pathway in EJ Cells. (**A**) Kae induces apoptosis in EJ cells. EJ cells were treated with indicated concentrations of Kae for 24 h. Apoptotic cells were determined by flow cytometry; (**B**) Summarized flow cytometry data of Kae-treated EJ cells; (**C**) Kae activated cleaved caspase-3. EJ cells were treated with indicated concentrations of Kae for 24 h; levels of procaspase-3 and cleaved caspase-3 were detected by Western blot; (**D**) Relative cleaved caspase-3 protein. Data are expressed as the mean ± SD from three independent experiments. *p < 0.05, **p < 0.01 *vs*. DMSO-only group.

To see whether Kae induced apoptosis in EJ cells, we performed flow cytometric analysis of annexin V- and propidine iodide (PI)-stained cells. EJ cells were treated with DSMO only or 20, 40, 80 or 160 μM of Kae for 24 h; apoptotic rates in Kae-treated groups were increased to 17.0%, 19.4%, 25.1%, 55.3% of the DMSO-only group, respectively (Figure 2A). This result indicated that Kae induces apoptosis in EJ cells in a dose-dependent manner (Figure 2B). As cleaved caspase-3 is an effector in apoptosis, we next detected cleaved caspase-3 in Kae-treated EJ cells with Western blot. Kae significantly decreased procaspase-3 and increased caspase-3 cleavage in a dose-dependent manner (Figure 2C,D). These results demonstrated that the activation of caspase-3 pathway is involved in Kae-induced apoptosis in EJ cells.

#### 2.1.3. Kae Suppresses Migration of EJ Cells

**Figure 3:**
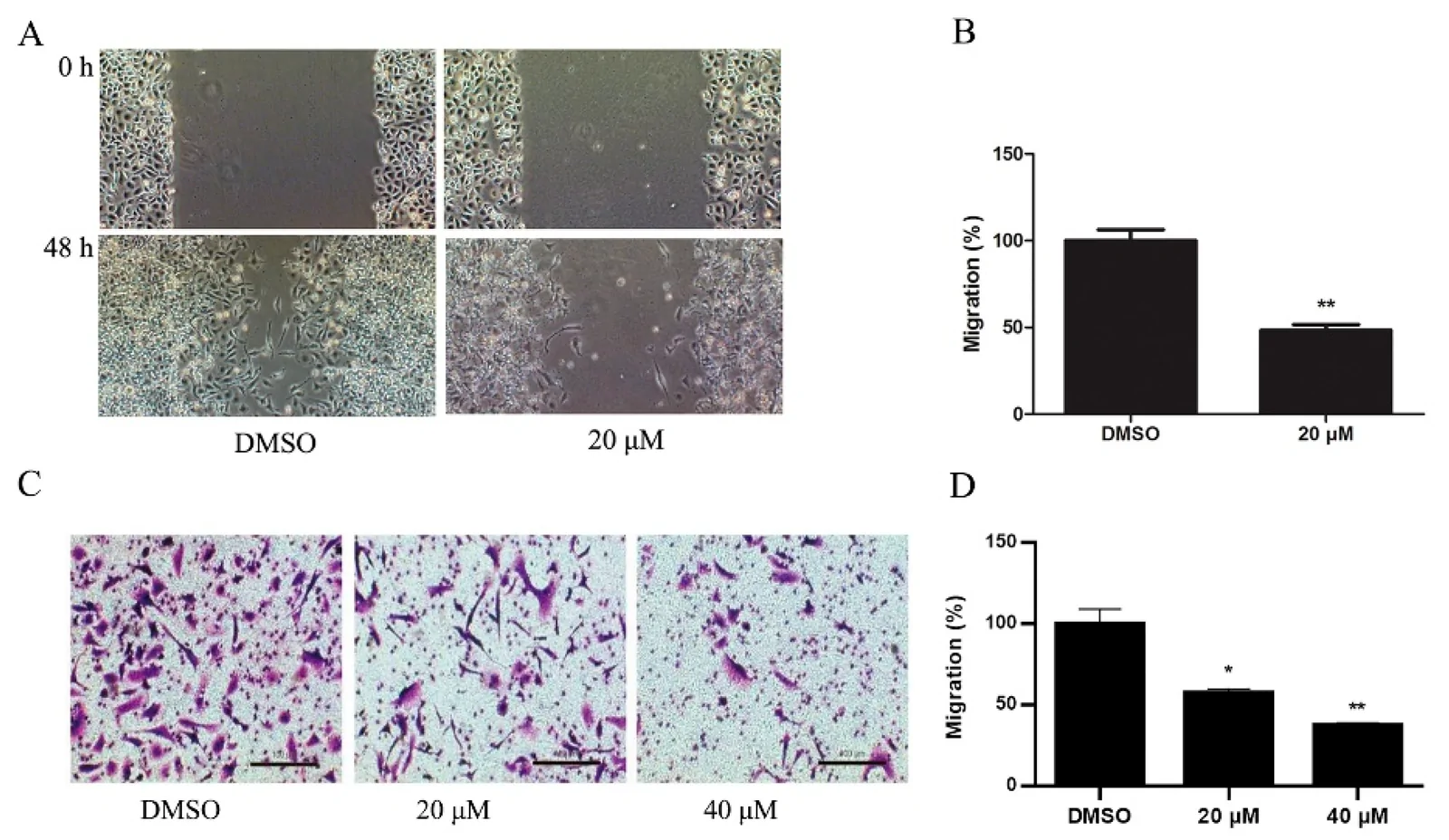
Kae suppresses migration of EJ cells. (**A**) Wound-healing assay in EJ cells treated with DMSO or 20 μM Kae for 48 h; (**B**) Migrated cells in wound-healing assay were counted under a microscope at ×200. Data from three independent experiments were analyzed and presented as the mean ± SD (** *p* < 0.01 vs. DMSO-only group); (**C**) Transwell cell migration assay in EJ cells with DMSO-only or 20 or 40 μM Kae for 48 h; (**D**) Migrated cells on the lower chamber membrane were counted. Data from three independent experiments were analyzed and presented as the mean ± SD. * *p* < 0.5, ** *p* < 0.01 vs. DMSO-only group.

To evaluate the effect of Kae on the motility of EJ cells, we performed a wound-healing assay. After incubating EJ cells with 20 μM Kae for 48 h, we found that the migrating cells in the treated group were significantly reduced, to 51.6% of DMSO-only controls (Figure 3A,B). This accords with the transwell migration assay, in which migrated cells in 20- and 40-μM Kae-treated groups were reduced to 37.1% and 59.3%, respectively, of the DMSO-only group (Figure 3C,D).

#### 2.1.4. Kae-Induced Apoptosis in EJ Cells Involves PTEN

**Figure 4:**
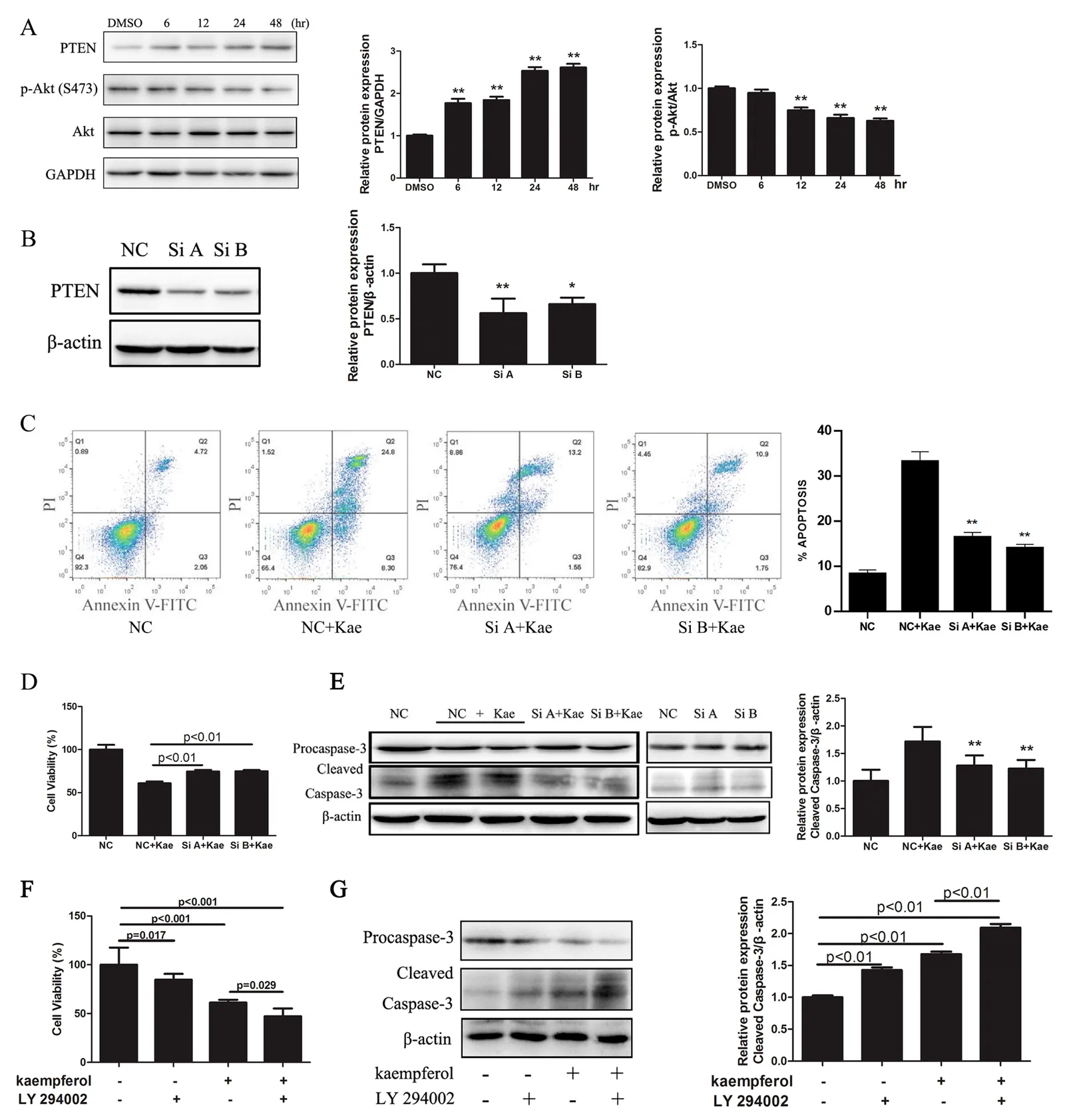
Kae-induced apoptosis in EJ cells involves PTEN. (**A**) Kae upregulates PTEN expression in a time-dependent manner. EJ cells were treated with 40 μM Kae for the indicated times. Levels of PTEN and Akt were detected by Western blotting. Data are expressed as the mean ± SD from three independent experiments (** *p* < 0.01 vs. DMSO-only group); (**B**) siRNA (Si A and Si B) inhibits PTEN expression; control siRNA was used as the negative control (NC) (* *p* < 0.05, ** *p* < 0.01 vs. NC); (**C**) PTEN siRNA inhibits Kae-induced apoptosis. PTEN siRNA-transfected EJ cells treated with 40 μM Kae for 24 h resulted in significantly decreased apoptosis, compared with NC siRNA-transfected cells treated with 40 μM Kae. Data represent the mean ± SD of three independent experiments (** *p* < 0.01 vs. NC + Kae group); (**D**) PTEN siRNA attenuates the growth-inhibiting effects of Kae. PTEN siRNA- or NC siRNA-transfected EJ cells were treated with 40 μM Kae for 24 h; cell viability was determined by the CCK-8 assay. Data shown are the mean ± SD of three independent experiments; (**E**) PTEN siRNA blocks Kae-induced cleavage of caspase-3. Western blots of cleaved caspase-3 in PTEN siRNA- or NC siRNA-transfected cells treated with 40 μM Kae for 24 h (** *p* < 0.01 vs. NC + Kae group); (**F**) LY294002 enhances Kae-inhibition of EJ cell growth. EJ cells were treated with 40 μM Kae, 20 μM LY294002 or both for 24 h. Cell viability was determined by the CCK-8 assay. Data shown are the mean ± SD of three independent experiments; (**G**) LY294002 enhances Kae-induced cleavage of caspase-3. EJ cells were treated with 40 μM Kae, 20 μM LY294002 or both for 24 h. Western blots of cleaved caspase-3 were performed.

We evaluated levels of PTEN and Akt in EJ cells treated with Kae and observed increased PTEN expression in a time-dependent manner in EJ cells treated with 40 μM Kae, whereas expression of Ser473-phosphorylated Akt (pAkt) was substantially decreased after 12 h of treatment with 40 μM Kae (Figure 4A). Together, these results indicate that Kae upregulates PTEN expression and inhibits pAkt (Ser473) in EJ cells.

Next, to explore the role of PTEN in Kae-induced apoptosis in EJ cells, we knocked down PTEN using two different siRNAs. PTEN siRNAs (Si A and Si B) successfully suppressed basal PTEN expression by 43.9% and 33.9%, respectively (Figure 4B). We then examined the effect of knocked-down PTEN expression on Kae-induced apoptosis in EJ cells using flow cytometry. Suppression of PTEN decreased Kae-induced EJ cell apoptosis by about 7.0% and 8.5% compared with controls (Figure 4C). Meanwhile, a cell viability assay showed that knocked-down PTEN expression attenuated the growth-inhibiting effects of Kae in EJ cell (Figure 4D). Moreover, Western blotting indicated that knocked-down PTEN expression prevented activation of caspase-3 induced by Kae (Figure 4E). Collectively, our results show that knocking down PTEN with siRNAs decreased apoptosis-induction by Kae in EJ cells, which implies that upregulation of PTEN accounts, at least in part, for apoptosis in EJ cells caused by Kae.

Finally, we suppressed Akt activity with a PI3K-specific inhibitor, LY294002, in Kae-incubated EJ cells. Cell growth was then assessed with the CCK-8 assay. Kae treatment combined with LY294002 inhibited growth more than did Kae alone (Figure 4F). Furthermore, Western blotting indicated that LY294002 enhanced Kae-induced activation of the cleavage of caspase-3 (Figure 4G). LY294002 enhanced Kae-inhibition of cell growth and Kae-induced cleavage of caspase-3, which implies that the PTEN/PI3K/Akt signaling pathway mediates Kae’s antitumor effects on EJ cells.

### 2.2. Discussion

Kaempferol, a dietary flavonoid widely presents in fruits and vegetables, has been shown to possess chemopreventive or chemotherapeutic properties for different cancers [10,19,20]. In this study, we assessed the antitumor effects of Kae on bladder cancer cells. We show, for the first time, that Kae inhibits proliferation, induces apoptosis and suppresses migration in EJ cells, and these effects are at least partially mediated by the PTEN/PI3K/Akt signaling pathway as a novel mechanism.

The most important characteristic of a cancer cell is the ability to sustain proliferation [21]. The cellular pathways that control proliferation in normal cells are disturbed in most cancers [22]. Thus, we first analyzed the effect of Kae on the viability of EJ and T24 cells. Our results clearly showed that Kae significantly inhibited proliferation of both cell lines in a dose-dependent manner, even at 20 μM in EJ cells after 24 h of treatment.

Because some anticancer drugs exert their antitumor effect through apoptosis [23,24], we investigated the effect of Kae on apoptosis in EJ cells. Treatment with Kae for 24 h resulted in significant apoptosis in EJ cells in a dose-dependent manner. Because apoptosis involves a cascade of proteolytic activity, mainly carried out by caspases [25], we investigated the protein expression of caspase-3, the executor of cell apoptosis. After treatment with Kae for 24 h, cleaved caspase-3 was upregulated in a dose-dependent manner. These results indicate that Kae-induced apoptosis in EJ cells involves caspase activation.

Metastasis, which is initiated by the migration and invasion of cells into the surrounding vasculature, is the primary cause of morbidity and mortality in cancer patients [26]. We therefore initially analyzed the effect of Kae on migration; our study is the first to demonstrate that Kae could inhibit the migratory properties of EJ cells. Consistent with our results, Kae also inhibits the migration and invasion of breast cancer and melanoma cells [27,28]. These observations, taken together, imply that Kae broadly suppresses the metastasis of many types of cancer cells.

PTEN is a potent tumor suppressor. Recent studies have shown that reduced PTEN protein expression is common in bladder cancer [29,30]. Therefore, exploitation of genes and drugs that regulate PTEN in tumors is an important strategy in treating cancer or overcoming resistance to anticancer drugs. In the present study, we found that Kae significantly increased PTEN expression in EJ cells. We therefore propose that Kae-induced EJ cell apoptosis involves increased PTEN expression. This hypothesis was confirmed by our results that show siRNA-suppressed PTEN expression attenuates the growth-inhibiting effects of Kae, blocks Kae-induced increases in cleaved caspase-3 protein and abolishes apoptosis induced by Kae in EJ cells. The data show that increased PTEN is at least partly responsible for Kae-induced apoptosis in EJ cells. Our finding suggests that Kae could be an alternative therapy for bladder cancer, based on its PTEN activation effect.

PTEN inhibits PI3K/Akt signaling, which is a central regulator of cell proliferation and apoptosis [31]. Wu X. *et al*. [32] reported that >50% of bladder cancers had significantly higher levels of phosphorylated Akt than normal controls. In Kae-treated EJ cells, we observed decreased Akt phosphorylation. Cells treated with a combination of Kae and LY294002 (a PI3K inhibitor) had more inhibited growth than did cells treated with Kae alone. Moreover, LY294002 enhanced Kae-induced cleavage of caspase-3. These results indicate that Kae inhibits PI3K/Akt signaling by upregulating PTEN. This is consistent with a previous study that showed Kae to induce apoptosis in leukemia cells via Akt inactivation [11]. Our finding implies that Kae inhibits the canonical PI3K/Akt pathway and its downstream effects. Activation of the PI3K/Akt pathway reportedly promotes chemoresistance in bladder cancer, whereas upregulation of PTEN and reduction of Akt phosphorylation restored doxorubicin sensitivity in bladder tumor cells [33]. This implies that Kae could help counter resistance when used with existing cancer therapies.

Garcia R *et al*. [34] reviewed the relationship between phyto-chemicals and bladder cancer using a dietary history questionnaire in 1999. This retrospective study concluded that the intake of specific flavonoids failed to decrease bladder cancer risk. We therefore hypothesize that Kae’s role in bladder cancer works through inhibition of tumor progression and metastasis, rather than tumorigenesis. Rajbhandari R. *et al*. [35] reported that Kae, a metabolite of cranberries, was found in rat urine after cranberry treatment in rats. This implies that Kae may exert anticancer effects on bladder cancer through actions in both blood and urine. In the present *in vitro* study, we find an impressive antineoplastic potential of Kae in bladder cancer, but the bioavailability of Kae is relatively low *in vivo*, because of first-pass metabolism in liver [36]. However, it is feasible to apply intravesical instillation to increase local drug concentration in bladder cancer tissue, and it is promising to utilize nanotechnology to increase Kae’s bioavailability, thus augmenting its anti-cancer properties.

## 3. Experimental Section

### 3.1. Reagents and Cell Culture

Kaempferol was purchased from Sigma-Aldrich (St. Louis, MO, USA). A stock solution of Kae was prepared in DMSO at 80 mM, stored at −20 °C and diluted in fresh media to the required concentration immediately before use. The concentration of DMSO used in the vehicle control is equal to that in the treatment group with the highest dose. LY294002 was purchased from Cell Signaling Technology (CST, Beverly, MA, USA). Cell culture reagents, such as fetal bovine serum (FBS), antibiotic mixtures, trypsin and phosphate-buffered saline (PBS), were purchased from Thermal Fisher Scientific (Schuylerville, NY, USA). EJ and T24 cell lines were obtained from the Institute of Urology, Peking University, and cultured in RPMI-1640 containing 10% FBS, 100 units/mL penicillin and 0.1 mg/mL streptomycin. Cells were maintained in an incubator at 37 °C with 5% CO_2_.

### 3.2. Cell Viability Assay

The effect of Kae on cell viability was measured using CCK-8 (Dojindo, Kumamoto, Japan). Cells were seeded in 96-well plates at 4 × 10^3^ cells/well, incubated overnight and then exposed to the indicated concentrations of Kae for 24 h and 48 h; 10 μL of CCK-8 reagent was then added to 100 μL fresh culture media per well. Plates were then incubated at 37 °C for 1.5 h. Optical densities were read at 450 nm.

### 3.3. Apoptosis Analysis

Apoptosis was analyzed by flow cytometry (FACSCalibur, BD, Franklin Lakes, NJ, USA), according to the protocol for the fluorescein isothiocyanate (FITC) Annexin V Apoptosis Detection Kit I (BD Biosciences, Franklin Lakes, NJ, USA). Cells were seeded at 1 × 10^4^ cells/mL in 60-mm culture plates for 24 h and then treated with medium containing various concentrations of Kae or DMSO for 24 h. Cells were then harvested using trypsin, washed twice with ice-cold PBS and resuspended in 1× binding buffer with 5 μL annexin V and 5 μL propidium iodide added. The mixture was incubated for 15 min at room temperature in the dark; 400 μL binding buffer was then added, and cells were analyzed immediately by flow cytometry.

### 3.4. Western Blot Analysis

EJ cells were cultured with serum-free medium for 6 h and then treated with various concentrations (0, 20, 40, 80 or 160 μM) of Kae in DMSO vehicle for 24 h. Total protein extracts were obtained by lysing cells in cold radioimmunoprecipitation assay (RIPA) buffer (20 mM Tris, 2 mM EDTA, 1% Triton X-100, 1% sodium deoxycholic acid and 0.1% sodium dodecyl sulfate) with 1% phenylmethanesulfonyl fluoride (PMSF). Equal amounts of proteins were separated on a 12% SDS-PAGE, blotted onto polyvinylidene fluoride (PVDF) membranes (Millipore, Bedford, MA, USA) and blocked with 5% nonfat milk in Tris-buffered saline (TBS)/Tween (0.05% Tween-20 in TBS). Blots were probed with procaspase-3, cleaved caspase-3, β-actin, PTEN, p-Akt (Ser473), Akt and glyceraldehyde-3-phosphate dehydrogenase (GAPDH) antibodies (CST, Beverly, MA, USA), respectively, and were then developed using a chemiluminescence kit (Millipore, Bedford, MA, USA) with G:BOX Chemi XL1. GENESys (Syngene, Cambridge, UK).

### 3.5. Wound-Healing Assay

EJ cells were seeded in 6 well plates and cultured to confluence. Cells were then scratched with a 1-mL pipette tip and washed three times with PBS. Medium containing 1% FBS and various concentrations of Kae was added to the wells. Wound areas were examined after 48 h of incubation under an OLYMPUS inverted microscope connected to a DXM1200 digital camera (Nikon, Tokyo, Japan). Migration ability was estimated by the number of migrated cells.

### 3.6. Transwell Cell Migration Assay

EJ cell migration was assayed using Transwell vessels (Corning, NY, USA) with 8-μm pore membranes. We incubated 100 μL of the cell suspension (1 × 10^4^ cells/mL) at 37 °C for 18 h in serum-free medium in the upper chambers with various concentrations of Kae and 500 μL of RPMI-1640 medium with 10% FBS in the lower chambers. After incubation, non-migrated cells on the top membrane surface were removed with a cotton bud. The membrane with migrated cells was fixed with 4% paraformaldehyde for 15 min and stained with 0.1% crystal violet for 20 min. Migrated cells were counted in 5 randomly selected high power fields (200×) under a microscope.

### 3.7. Small Interfering RNA-Mediated Knockdown of PTEN

To downregulate PTEN expression, siRNA duplexes that specifically target human PTEN (A. sense: 5′-CCAGUCAGAGGCGCUAUGUTT-3′; antisense: 5′-ACAUAGCGCCUCUGACUGGTT-3′; B. sense: 5′-CCACCACAGCUAGAACUUATT-3′; antisense: 5′-UAAGUUCUAGCUGUGGUGGTT-3′) were synthesized and purchased from Genepharma (Shanghai, China); siRNA duplexes with non-specific sequences were used as the negative control. SiRNA transfection was carried out with Lipofectamine RNAiMAX (Invitrogen, Carlsbad, CA, USA) at a final concentration of 100 nmol/L, according to the manufacturer’s protocol. At 48 h post-transfection, cells were treated with or without 40 μM Kae for another 24 h. Cell viability was detected by CCK-8. Apoptosis was analyzed by flow cytometry. Cell lysates were subjected to immunoblot for cleaved caspase-3.

### 3.8. Statistical Analysis

All replications of an experiment were averaged, and the mean values from different experiments were pooled for statistical analysis. One-way ANOVA followed by least significant difference test (LSD test) or *t*-test was performed to determine the differences between groups. Analyses used SPSS statistics 17.0 software (SPSS Inc., Chicago, IL, USA, 2008). *p* < 0.05 was considered to be significant.

## 4. Conclusions

In summary, we have shown for the first time that Kae can inhibit proliferation, induce apoptosis and suppress migration in human bladder cancer cells and mediates an apoptotic mechanism that involves the upregulation of PTEN and inhibition of Akt phosphorylation. These findings indicate that Kae has a therapeutic role for bladder cancer, as a preventative or as an enhancement for existing treatments. Moreover, its effects on PTEN and Akt suggest that Kae could be an alternative medicine for bladder cancer.
