# Correction: Xie et al. Kaempferol Promotes Apoptosis in Human Bladder Cancer Cells by Inducing the Tumor Suppressor, PTEN. *Int. J. Mol. Sci.* 2013, *14*, 21215–21226

**DOI:** 10.3390/ijms27208989

**Published:** 2026-10-10

**Authors:** Feng Xie, Ming Su, Wei Qiu, Min Zhang, Zhongqiang Guo, Boxing Su, Jie Liu, Xuesong Li, Liqun Zhou

**Affiliations:** 1 Department of Urology, Peking University First Hospital & the Institute of Urology, Peking University, Beijing 100034, China; xiefengjoy@126.com (F.X.); qiuwei618@163.com (W.Q.); pkugzq@gmail.com (Z.G.); 05202154@163.com (B.S.); soloriver@126.com (J.L.); pineneedle@sina.com (X.L.); 2 National Urological Cancer Center, Beijing 100034, China; 3 Sino-German Laboratory for Molecular Medicine, State Key Laboratory of Cardiovascular Disease, FuWai Hospital & Cardiovascular Institute, Chinese Academy of Medical Sciences, Peking Union Medical College, Beijing 100037, China; suming28@163.com; 4 Department of Urology, Shanxi Medical University First Hospital, Taiyuan 030001, China; 15201286472@163.com

In the original publication [1], there were two mistakes in Figure 3C and Figure 4C as published. The transwell image was misplaced in Figure 3C and the flow cytometry result was misplaced in Figure 4C. The corrected Figure 3 and Figure 4 appear below.

**Figure 3:**
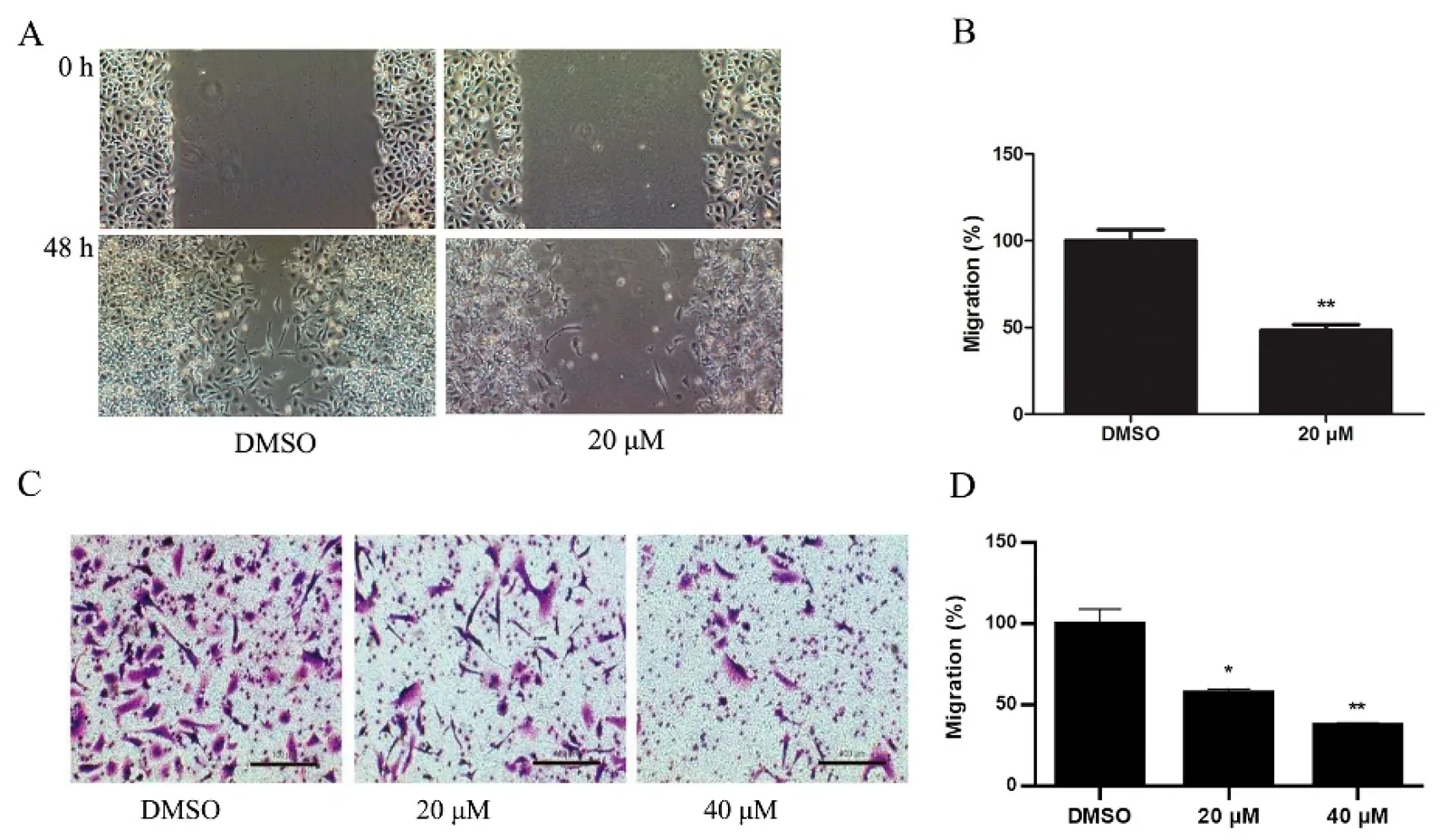
Kae suppresses migration of EJ cells. (**A**) Wound-healing assay in EJ cells treated with DMSO or 20 μM Kae for 48 h; (**B**) Migrated cells in wound-healing assay were counted under a microscope at ×200. Data from three independent experiments were analyzed and presented as the mean ± SD (** *p* < 0.01 vs. DMSO-only group); (**C**) Transwell cell migration assay in EJ cells with DMSO-only or 20 or 40 μM Kae for 48 h; (**D**) Migrated cells on the lower chamber membrane were counted. Data from three independent experiments were analyzed and presented as the mean ± SD. * *p* < 0.5, ** *p* < 0.01 vs. DMSO-only group.

**Figure 4:**
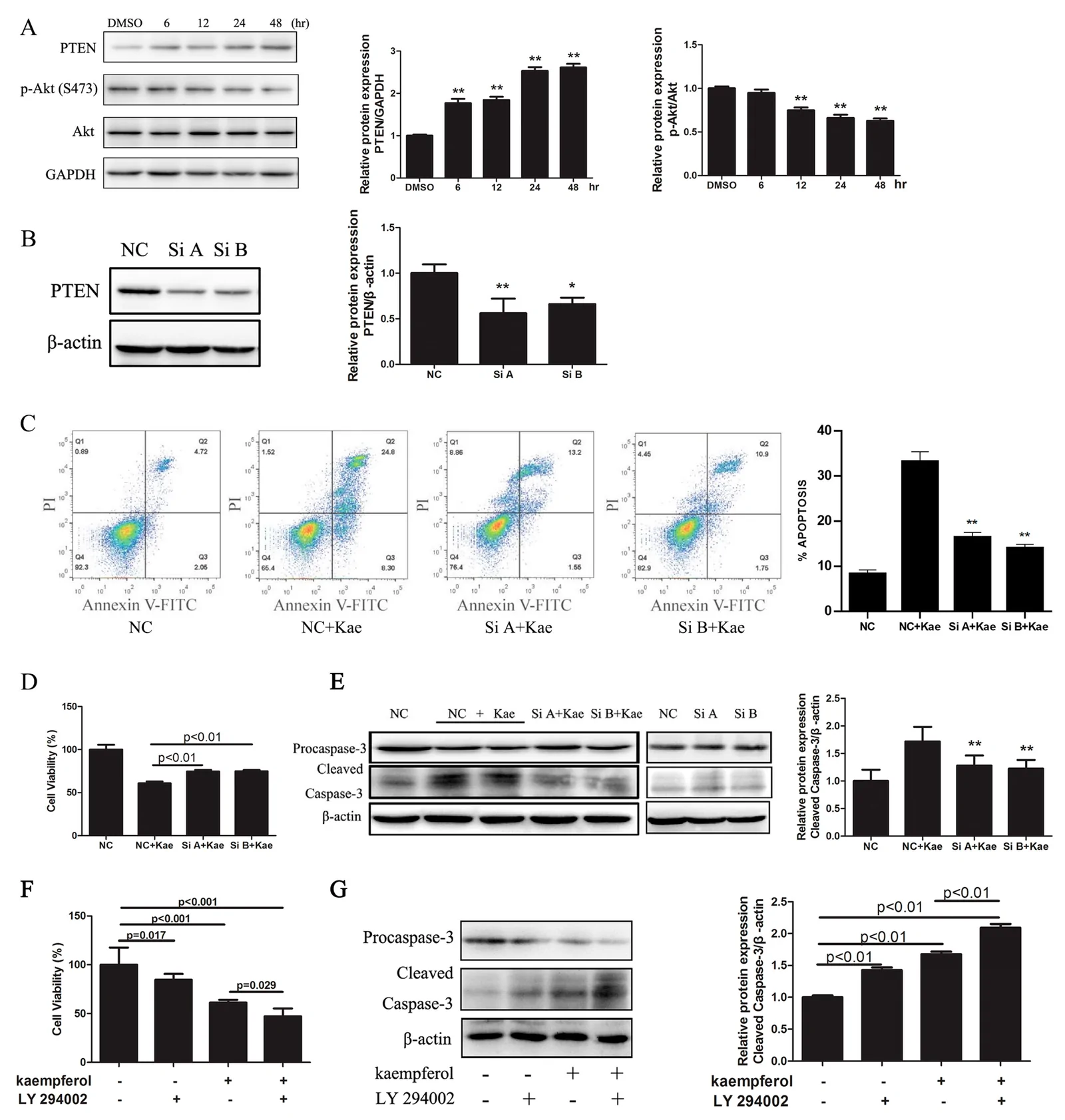
Kae-induced apoptosis in EJ cells involves PTEN. (**A**) Kae upregulates PTEN expression in a time-dependent manner. EJ cells were treated with 40 μM Kae for the indicated times. Levels of PTEN and Akt were detected by Western blotting. Data are expressed as the mean ± SD from three independent experiments (** *p* < 0.01 vs. DMSO-only group); (**B**) siRNA (Si A and Si B) inhibits PTEN expression; control siRNA was used as the negative control (NC) (* *p* < 0.05, ** *p* < 0.01 vs. NC); (**C**) PTEN siRNA inhibits Kae-induced apoptosis. PTEN siRNA-transfected EJ cells treated with 40 μM Kae for 24 h resulted in significantly decreased apoptosis, compared with NC siRNA-transfected cells treated with 40 μM Kae. Data represent the mean ± SD of three independent experiments (** *p* < 0.01 vs. NC + Kae group); (**D**) PTEN siRNA attenuates the growth-inhibiting effects of Kae. PTEN siRNA- or NC siRNA-transfected EJ cells were treated with 40 μM Kae for 24 h; cell viability was determined by the CCK-8 assay. Data shown are the mean ± SD of three independent experiments; (**E**) PTEN siRNA blocks Kae-induced cleavage of caspase-3. Western blots of cleaved caspase-3 in PTEN siRNA- or NC siRNA-transfected cells treated with 40 μM Kae for 24 h (** *p* < 0.01 vs. NC + Kae group); (**F**) LY294002 enhances Kae-inhibition of EJ cell growth. EJ cells were treated with 40 μM Kae, 20 μM LY294002 or both for 24 h. Cell viability was determined by the CCK-8 assay. Data shown are the mean ± SD of three independent experiments; (**G**) LY294002 enhances Kae-induced cleavage of caspase-3. EJ cells were treated with 40 μM Kae, 20 μM LY294002 or both for 24 h. Western blots of cleaved caspase-3 were performed.

The authors state that the scientific conclusions are unaffected. This correction was approved by the Academic Editor. The original publication has also been updated.
